# Langya henipavirus (LayV) as an emerging zoonotic disease: a mini-review

**DOI:** 10.1016/j.nmni.2025.101643

**Published:** 2025-10-08

**Authors:** Maryam Shafaati, Milad Zandi

**Affiliations:** a Research Center for Antibiotic Stewardship and Antimicrobial Resistance, Infectious Diseases Department, Imam Khomeini Hospital Complex, Tehran University of Medical Sciences, Tehran, Iran; b Center for Communicable Disease Control, IPC/AMR Office, Ministry of Health and Medical Education, Tehran, Iran; c Iranian Society for Virology, Deputy for Education, Ministry of Health and Medical Education, Tehran, Iran

**Keywords:** Langya henipavirus, Henipavirus, Zoonotic

## Abstract

Henipavirus is one of the genera in the *Orthoparamyxovirinae* subfamily, which includes several emerging viruses that pose a major public health threat. The predominant members of the virus genus, Hendra and Nipah viruses, are extremely virulent zoonotic viruses that cause neurological and respiratory infections and outbreaks in humans. The recently discovered Langya henipavirus, a new henipavirus phylogenetically related to Mojiang henipavirus (MojV), has been associated with febrile illness in patients from China who are mainly agricultural workers. Active surveillance must be conducted worldwide in an open and collaborative manner to reduce the likelihood of this new virus causing a health crisis. More research is needed to address the remaining difficulties.

## 1 Introduction

It is estimated that 70 % of emerging infectious diseases (zoonotic or zoonotic in origin) are transmitted to humans through contact with animals as the human population increases in the natural environment [1]. The key to preventing pandemics lies in strengthening a disease surveillance system based on a public health approach and early detection of outbreaks caused by new viruses. Climate change and loss of biodiversity have increased the potential for “zoonotic spillover,” the spread of viruses from animals to humans. Pandemics are more likely in the future because of climate change, which is exacerbated by deforestation, extinction, increased human immigration, and animal contact [2,3]. In the past and in the present, emerging viruses have negatively affected public health. Most of them originate from wild animals such as bats, rodents, nonhuman primates, and mosquitoes. The following viruses are new or have reemerged: Arboviruses, Coronaviruses, Arenaviruses, and Paramyxoviruses.

Paramyxoviruses have evolved in a complex manner, indicating that they have a much broader spectrum of genomic organization than previously thought. Epidemiologically, there is a need for surveillance and tracking of the emergence of emerging viral infections, particularly those belonging to the henipavirus genus. Langya henipavirus (LayV), a previously unknown and phylogenetically distinct virus belonging to the henipavirus genus, was recently identified in China. Based on the limited evidence available, the risk of infection for frequent travelers worldwide is considered low. LayV shares genetic similarities with Nipah and Hendra viruses, which are also henipaviruses and can cause respiratory infections that can be fatal. More likely, the virus is transmitted from animals to humans. Among the 25 different species of small wild animals, shrews are the only species that have tested positive for the virus' RNA so far. These results suggest that shrews are a potential natural reservoir for LayV virus [4]. The henipavirus genus contains two extremely virulent zoonotic viruses (Hendra and Nipa viruses) that are of great public health importance. Infections with Hendra and Nipah henipaviruses occur only in Australia and Southeast Asia. Most henipavirus viruses are transmitted by bats. However, there are some exceptions that have been mentioned, including emerging viruses that use rodents and shrews as reservoir species. There is limited evidence of the spread of these viruses in the Americas. Analysis of limited epidemiologic data revealed that human-to-human transmission is uncommon and that multiple spillover occurrences are more likely to result in human cases [5,6]. These findings increase the likelihood that mammals other than bats and rodents are carriers of henipaviruses. They also demonstrate how these viruses can evolve into new infections in humans regardless of the taxonomic classification of the animals that serve as reservoirs. Because of the importance of emerging viruses, we focus on a new henipa-like virus called Langya henipavirus (LayV) that was recently identified in China. This commentary highlights the potential impact of LayV, assesses the current problem, and presents effective containment measures to reduce it.

## 2 Methods

This mini-review was compiled through a comprehensive search of scientific literature focusing on LayV and other relevant henipaviruses. The primary goal was to synthesize current knowledge regarding LayV's discovery, characteristics, transmission, public health implications, and potential control strategies. Searches were conducted across major scientific databases, including PubMed, Scopus, and Google Scholar.

The search strategy employed various keywords and their combinations, such as “Langya henipavirus,” “LayV,” “Henipavirus,” “Nipah virus,” “Hendra virus,” “zoonotic,” “emerging virus,” “paramyxovirus,” “transmission,” “reservoir host,” “diagnosis,” “treatment,” and “public health.” Papers were selected based on their direct relevance to these themes, with an initial screening of titles and abstracts followed by full-text review. Priority was given to original research articles, comprehensive review articles, and reports from established health organizations that provided key insights into the epidemiology, pathogenesis, and broader impact of these viruses.

### 2.1 The characterization of newly detected henipavirus

The Paramyxoviridae are one of the largest known families of single-stranded negative sense RNA viruses after the Rhabdoviridae. The Paramyxoviridae family may evolve into new viruses for humans. This subfamily includes several important animal pathogens such as bovine plague virus and human pathogenic viruses that can infect humans, and other animals, including measles, mumps, parainfluenza viruses, and Hendra and Nipa viruses [1]. Recently, many new orthoparamyxoviruses have been identified, and some of them are considered pathogenic to humans and animals. LayV is an enveloped virus with a single-stranded RNA genome that is negatively oriented, similar to other viruses in the genus Henipavirus of the family Paramyxoviridae [2].

The *Paramyxoviridae* family now includes four subfamilies, 17 genera, and 86 species (the largest subfamily is *Orthoparamyxovirinae*).

**Fig. 1:**
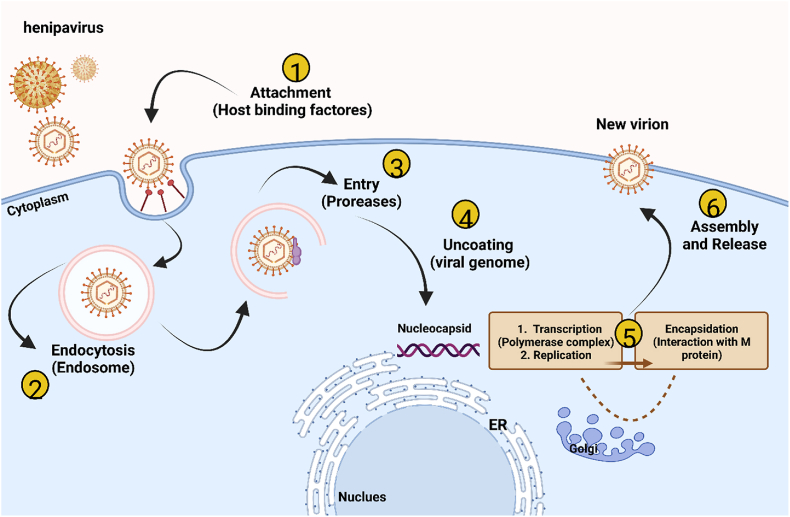
Cell cycle and replication of Henipavirus. At every stage of the viral life cycle, including attachment, uncoating, genomic replication, protein expression, viral assembly, and release, virus-host interactions occur. Many of the contributing components are yet unknown**.** This figure was produced using the BioRender.

This is a large group of negative-sense RNA viruses with non-segmented genomes ranging in size from 14.296 to 20.148 nucleotides. A nucleocapsid protein (N), a matrix protein (M), a fusion protein (F), a receptor binding protein (RBP), and a phosphoprotein (P) interact with a polymerase protein (L) to form the RNA-dependent RNA polymerase complex. All appear to be proteins encoded by genomes [3,4] (Fig. 1).

Blood vessel endothelial cells are the primary target cells in the late stages of Henipavirus infection in humans, which can lead to vasculitis, vasculitis-related thrombosis, and vascular microinfarcts in the central nervous system (CNS) and other organs such as the lungs, spleen, and kidneys. The parenchymal cells of the CNS are also infected, and these cells together contribute significantly to the pathogenesis of henipaviruses. Although the exact route of infection in humans is uncertain, previous studies in a number of laboratory animal models have shown that intranasal exposure does indeed cause infection, suggesting that the respiratory system is one of the first organs in which a virus replicates. In addition, both Nipa and Hendra viruses have been found in respiratory secretions from humans and animals, underscoring the importance of the respiratory system for virus replication and potential transmission. Henipavirus infection in humans is characterized by an influenza-like illness that can lead to pneumonia or acute respiratory distress syndrome (ARDS) [5,6].

Globally, Nipah virus is of greater concern, and Bangladesh has typically been the site of outbreaks. There are various degrees of severity of Nipah virus infection, ranging from extremely mild to fatal encephalitis. The first outbreaks in Malaysia and Singapore were reported by people who had close contact with pigs. Recent epidemics of the Nipah virus are thought to have been caused by food contaminated with the urine or saliva of infected bats. The virus is believed to spread from person to person, primarily among individuals living in the same household. In 1994, 14 horses and their trainer in Queensland died from the Hendra virus. Since then, several outbreaks in horses have been documented, which are generally believed to be spillover infections from flying foxes. Four of the seven Australians infected with the Hendra virus have died. Most of those who contracted the virus were veterinarians who had contact with sick horses [[7], [8], [9]]. Currently, henipavirus outbreaks are limited to Australia and Southeast Asia [10]. As a result, there are fewer studies on viruses in this genus conducted outside of these regions. However, important serological surveys and phylogenetic research conducted in Africa on both humans and animals have also revealed the presence of henipavirus.

The transmission path of LayV is currently unknown, and it is unclear whether shrews spread the disease directly or through an intermediary host [17]. We believe that LayV is transferred either directly to people or through an intermediary animal because there are no obvious linkages between the cases [18,19]. As there were no clusters, there was no evidence that LayV was disseminating from one person to another. Only 35 additional individuals have contracted the disease since 2018, indicating that there is no relation between any of the cases. Currently, there are no indications that the virus can spread from one person to another [4], and no signs suggest that the patients were part of a larger epidemiological pattern. The fact that the majority of the affected individuals are farmers supports the theory of sporadic zoonotic transmission [20].

Public health concerns should be closely monitored for potential threats, as this new virus is still not well understood and reported cases are likely just the beginning (20–22). It should be noted that the virus is closely related to two other human henipaviruses (Hendra and Nipah), which are known to cause respiratory infections and even death, with Nipah having a mortality rate of 90 %. Although the virus has a low probability of spreading to humans, there is no need to panic, as no cases of fatal or extremely serious LayV infections have been reported in people known to be infected with the virus.

### 2.2 The Langya henipavirus (LayV)

Most of the species in the Henipavirus genus are transmitted by bats. In Africa, the fruit bat Eidolon helvum, from which the genome of the Ghanaian bat henipavirus was first discovered, is believed to play a significant role. In Asia and Australia, bats of the Pteropus genus are the primary hosts [11]. The Mojiang henipavirus (MojV), which is suspected of causing human disease in China, was considered an exception due to its link to Rattus flavipectus rats [12,13]. Reports suggest that the Mojiang henipavirus enters human cells using a different method than the Hendra and Nipah viruses employ.

New henipaviruses have been primarily identified in insects of the genus Crocidura in China, South Korea, Belgium, and Guinea [4]. The henipavirus Langya virus (LayV) is a member of the Paramyxoviridae family (Table 1). Unlike other henipaviruses that are exclusively found in bats, its closest relative is the Mojiang henipavirus. LayV is also closely related to the Nipah virus and the Hendra virus, and it causes disease in humans. It is important to determine whether human-to-human transmission is possible for any newly discovered zoonotic virus [13]. Although Zhang et al. [14] reported that shrews are a natural reservoir for henipaviruses, these viruses have a wide range of hosts. Further research is needed to fully understand the host and reservoir species. However, if LayV spreads easily from person to person, it would be ubiquitous. During metagenomic analysis and virus isolation, it was found and isolated in a throat swab sample from a 53-year-old patient. From April 2018 to August 2021, there were 35 reported cases of Langya henipavirus (LayV) infection in the eastern Chinese provinces of Shandong and Henan. The virus appears to have primarily affected farmers, and there is no evidence of any association between the cases. Symptoms of LayV infection range from severe pneumonia to coughing, with common symptoms including high body temperature, fatigue, muscle aches, myalgia, nausea, and vomiting.

**Table 1:**
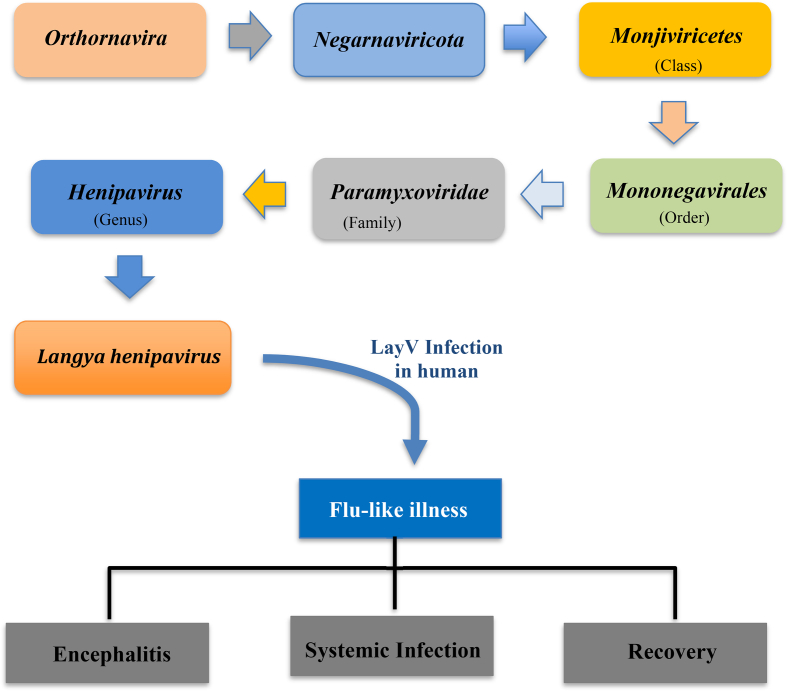
Classifying of the Langya henipavirus (LayV) and pathogenesis in humans.

At present, there are no reliable vaccines or licensed therapies available for henipaviruses such as the Langya virus. Ribavirin could be a potential treatment for viral infections when no other options are available. Ribavirin has shown success in treating the Hendra and Nipah viruses. Chloroquine, a malaria medication, is another treatment option for these two conditions. Therefore, in the absence of other treatment options, these two treatments may potentially help prevent the transmission of the Langya virus.

### 2.3 Symptoms and complications, and origin

By conducting sentinel surveillance of febrile cases with a history of animal contact in eastern China (Shandong and Henan provinces), 35 patients with acute LayV infection were identified. The patients frequently presented with symptoms such as fever, exhaustion, cough, anorexia, myalgia, nausea, headaches, and vomiting. 85 % of them were engaged in agricultural work. There have been no fatalities linked to the spread of LayV, which may indicate a low prevalence of infection. However, we cannot rule out the possibility of human cases occurring before 2019 or a larger geographical circulation of the virus due to the lack of specificity of the documented signs and symptoms. Most of the reported symptoms appear to be mild, and it is unknown how long patients have been experiencing them. Rare reports of potentially serious consequences, such as pneumonia, and abnormalities in liver and renal function have been documented. However, there is no information available on the severity of these abnormalities, whether hospitalization was required, or if any cases resulted in fatalities [14,15].

Surprisingly, local farmers were found to be the most affected by LayV, and there appears to be no connection between the cases. Coughing and acute pneumonia are just a few of the symptoms. Although LayV-infected individuals almost always exhibited fever, and respiratory symptoms such as cough and tiredness, no fatalities have been reported (22). Among the 26 patients with LayV infection, four pneumonia patients had the highest viral loads in their serum and throat swabs, indicating that the infection might spread through the blood. The novel LayV infection is expected to cause high rates of gingival bleeding, which is consistent with the thrombocytopenia condition associated with it. Like individuals infected with the Nipah virus, LayV patients experienced thrombocytopenia, pneumonia, leukopenia, and decreased liver/kidney functions. These laboratory results are also observed in COVID-19, although less frequently. Although not identical to mpox, the clinical symptoms match those of COVID-19. In areas with high COVID-19 transmission rates, this infection can easily be overlooked. Therefore, it is essential to evaluate individuals in China and surrounding areas who have a history of animal interaction, exhibit symptoms of fever and respiratory disease, but test negative for COVID-19 [16].

To determine if the virus originated from animals, antibodies against LayV were searched for in the blood of 25 species of small wild animals and animals that shared the same habitats as the infected individuals. In a serological study of domestic animals, this virus was found to be present in 2 % of goat sera and 5 % of dog sera [17,18]. However, higher levels of viral RNA were detected in Crocidura lasiura shrews when serum and viral RNA samples from wild rodents, including shrews, were analyzed. No epidemiological links were discovered among the examined cases. The fact that a large number of patients were farmers with more frequent animal contact than the general population supports the hypothesis of human-to-animal transmission. There is no evidence human to human transmission [19]. However, it is impossible to rule out this scenario. Further studies are needed to fully understand the modes of LayV transmission [14].

Because new diseases may emerge at any time, there is an urgent need for new and effective treatments for viral infections in high-risk epidemiological areas. Active surveillance must be conducted in an open and helpful manner on a global scale to reduce the likelihood that a new virus will cause a health catastrophe. Further studies are needed to address remaining concerns [20].

## 3 Conclusion and perspective

Because the discovery of this virus of the genus Henipavirus has not been previously reported, it underscores the continuing risk of the emergence of new infections. These conclusions are based on a limited sample size, and further analysis and study are needed to fully understand the epidemiologic and microbiologic characteristics of the virus and infection. For early response to potential pandemics, surveillance of pathogen emergence remains a key technique. Much more research is needed to understand the critically expanded surveillance and control measures, identify LayV hosts and reservoir animal species, and adopt a unified public health approach to reduce animal-human contacts.

## CRediT authorship contribution statement

**Maryam Shafaati:** wrote the first draft of the manuscript and drawn the figure.1. **Milad Zandi:** Writing – review & editing, Conceptualization.

## Consent to participate

*Not applicable* **.**

## Consent to publish

*Not applicable* **.**

## Ethics approval

*Not applicable* **.**

## Funding

The authors declare that no funds, grants, or other support were received during the preparation of this manuscript.

## Declaration of competing interest

The authors declare that they have no known competing financial interests or personal relationships that could have appeared to influence the work reported in this paper.
